# Emerging Role of Neutrophils in the Thrombosis of Chronic Myeloproliferative Neoplasms

**DOI:** 10.3390/ijms22031143

**Published:** 2021-01-24

**Authors:** Francisca Ferrer-Marín, Ernesto José Cuenca-Zamora, Pedro Jesús Guijarro-Carrillo, Raúl Teruel-Montoya

**Affiliations:** 1 Hematology and Medical Oncology Department, Hospital Universitario Morales-Meseguer, Centro Regional de Hemodonación, IMIB-Arrixaca, 30120 Murcia, Spain; ernestojose.cuenca@um.es (E.J.C.-Z.); raulteruelmontoya@hotmail.com (R.T.-M.); 2 CIBERER CB15/00055, 28029 Murcia, Spain; 3 Grade of Medicine, Faculty of Health Sciences, Catholic University of Murcia (UCAM), 30107 Murcia, Spain; pedrojesus.guijarro@gmail.com

**Keywords:** myeloproliferative neoplasms, neutrophils, thrombosis, NETs

## Abstract

Thrombosis is a major cause of morbimortality in patients with chronic Philadelphia chromosome-negative myeloproliferative neoplasms (MPN). In the last decade, multiple lines of evidence support the role of leukocytes in thrombosis of MPN patients. Besides the increase in the number of cells, neutrophils and monocytes of MPN patients show a pro-coagulant activated phenotype. Once activated, neutrophils release structures composed of DNA, histones, and granular proteins, called extracellular neutrophil traps (NETs), which in addition to killing pathogens, provide an ideal matrix for platelet activation and coagulation mechanisms. Herein, we review the published literature related to the involvement of NETs in the pathogenesis of thrombosis in the setting of MPN; the effect that cytoreductive therapies and JAK inhibitors can have on markers of NETosis, and, finally, the novel therapeutic strategies targeting NETs to reduce the thrombotic complications in these patients.

## 1. Introduction

Philadelphia chromosome-negative chronic myeloproliferative neoplasms (MPN) i.e., polycythemia vera (PV), essential thrombocythemia (ET), and myelofibrosis (MF), are clonal disorders of hematopoietic stem cells (HSC) that are characterized by a proliferation in the bone marrow (BM) of one or more myeloid lines. They share clinical features (e.g., splenomegaly, thrombotic complications, risk of leukemic transformation), and a common molecular basis: the dysregulation of the JAK-STAT pathway [1], which confers a hypersensitivity of HSC to the action of growth factors and cytokines, and, as a result, an overproduction of blood cells [2,3]. Abnormalities in blood cells are not only quantitative changes (leukocytosis, erythrocytosis, and thrombocytosis) but also qualitative alterations that induce the switch of these cells from a resting to a procoagulant phenotype [4,5]. Thus, thrombosis is one of the major causes of morbidity and mortality in MPN patients.

The pathogenesis of thrombosis in MPN is complex and multifactorial, and derives from the interaction of patient-specific factors (i.e., age, history of thrombosis, cardiovascular risk factors), and the disease itself. The thrombogenesis process seems to derive from the interaction of cellular (hyperviscosity, increase in red blood cell level, platelet and leukocytes cells activation) and plasma factors (microparticles, resistance to activated protein C) with the response of the endothelial cells to the inflammatory cytokines and mediators released by the neoplastic cells [4,5].

The complexity of the human MPN condition is amplified by co-operating mutations in myeloid genes that often accompany the p.V617F mutation in Janus Kinase 2gene (JAK2V617F) [6,7,8]. Among this array of factors, a mounting body of clinical and biological evidence supports the role of leukocytes in the thrombosis of MPN patients. Indeed, leukocytosis is a risk factor for thrombosis in PV and ET [9,10,11,12]. In this section, we will review the role of leukocytes, and more specifically, neutrophils, in thrombotic complications associated with MPN.

## 2. Thrombotic Complications in MPN Patients

The major thrombotic complications in MPN represent an important clinical problem due to their high morbidity, the complexity of their management and their associated mortality. The appearance of a thrombosis implies a stratification of high thrombotic risk of MPN patients and determines the beginning or optimization of cytoreductive treatment and the use of antiplatelet therapy or anticoagulant as secondary prophylaxis. Thrombotic events in MPN patients can range from microvascular thrombosis (erythromelalgia, migraine, vertigo, amaurosis fugax) to thrombosis in arterial (stroke, angina, infarction, peripheral arterial thrombosis) or venous territory (visceral, sinus, deep vein thrombosis -DVT-, pulmonary embolism). Thrombotic events often occur at the time of diagnosis (11–25% of ETs and 12–39% of PVs). In general, the most frequent thrombosis are arterial ones, particularly strokes, although acute coronary syndromes are the most frequent cause of death [2]. 

In the European Collaboration on low-dose Aspirin study (ECLAP), in patients with PV, the cumulative incidence of fatal and non-fatal thrombosis was 5.5 events per 100 patients/year [13]. The Gruppo Italiano Studio Policitemia followed 1213 patients for as long as 33 years (mean follow-up of six years) and found thrombotic events in 19% of patients during follow-up [14]. More recent studies, however, show lower rates of thrombosis, possibly due to the better control of cardiovascular risk factors and the better use of cytoreductors. In a study addressed by the International Working Group for MPN Research and Treatment (IWG-MRT) that included 1545 PV patients, the incidences of postdiagnosis arterial and venous thrombosis were 12% and 9%, respectively [15]. In the same line, the prospective randomized study (CYTO-PV) reported that total cardiovascular events occurred in 7.75% of patients [16]. In ET, the estimated range of thrombosis is 2–4% patient/year, with the frequency of arterial thrombosis being double that of venous thrombosis [11]. In primary MF, the incidence is similar to that of ET (2.33 events/person/years). Among venous thrombosis occurring in unusual sites, MPN is the most frequent cause of splenic vein thrombosis, accounting for 50% of cases of Budd-Chiari Syndrome (hepatic venous thrombosis) and 25% of portal vein thrombosis [17]. The brain is another uncommon place of venous thrombosis in MPN. JAK2 was reported mutated in the 1.7–6.5% of cerebral venous thrombosis patients [18,19].

Finally, the vascular complications of patients with MPN are not limited only to thrombosis. The anticoagulant or antiplatelet therapy that these patients often receive, along with other factors (such as acquired von Willebrand syndrome (AVWS) due to excessive thrombocytosis or the presence of esophageal varicose veins due to portal hypertension), increase the risk of bleeding. Consequently, the range of major bleeding varies from <1 to 8% depending on the series [20,21].

## 3. Thrombotic Risk Factors in MPN Patients

On the one hand, we can list clinical factors, such as age ≥60 and history of thrombosis as independent predictors of thrombosis in patients with MPN [11,13]. Thus, the absence of both identifies low-risk patients. In contrast, according to the recommendations of the European Leukemia Net (ELN) [22], the presence of either of them represents an indication to initiate cytoreductive treatment. With regard to the influence of cardiovascular risk factors (hypertension, hyperlipemia, obesity and diabetes), the studies carried out have had discrepant results [4]. In the recently developed International Prognostic System of Thrombosis for patients with World Health Organization (WHO) ET-criteria (IPSET), the cardiovascular risk factors are a significant and independent risk variable [23]. On the other hand, there are biological factors. Thus, the CYTO-PV study demonstrated that maintaining the hematocrit below 45% reduces the risk of thrombosis and death of cardiovascular origin compared to those patients who maintained the hematocrit levels between 45–50% [16]. In contrast, no study has shown a correlation between the number of platelets and the risk of thrombosis [4]. In fact, probably due to the binding of vWF to platelets, and consequently, to the depletion of large multimers (AVWS), extreme thrombocytosis (≥1500 × 10^9^/L) increases the bleeding risk [4,5].

Concerning leukocytosis, numerous studies have shown its relevance as a thrombotic risk factor (especially of arterial thrombosis) in both PV and ET [9]. The first observation came from a Mayo Clinic study of 322 patients with ET where the incidence of total (i.e., arterial and venous) thrombotic events occurring at diagnosis or during the follow-up was significantly higher in patients with a leukocyte count of 15 × 10^9^/L or higher. Subsequent expanded studies from the same group in low-risk patients with either ET or PV confirmed the association between leukocytosis and thrombosis (both arterial and venous) at diagnosis, but not during the follow-up [24,25]. The Italian group reported, by contrast that WBC count, at the time of the first thrombotic event, predicted recurrent arterial thrombosis in low-risk MPN patients [26]. In one of the largest epidemiologic studies in PV, the ECLAP study that included more than 1600 patients, baseline leukocytosis >15 × 10^9^/L, as opposed to <10 × 10^9^/L, predicted myocardial infarction but not venous thrombosis [27]. Overall, due to the retrospective nature of all these studies, highly heterogeneous, the question regarding the association between persistent leukocytosis and risk of thrombosis is unresolved. A recent meta-analysis of articles published in the last 12 years addressing the issue included more than 30,000 patients with ET or PV [12]. In the majority of studies included in this meta-analysis, the white blood cell (WBC) count was only measured at diagnosis or at the time of the enrollment, and overall thrombosis was evaluated without differentiating between arterial and venous events. Cutoffs used for the definition of leukocytosis ranged differently between studies and between diseases (ET (8.4–15.0 × 10^9^/L); PV (9.5–25.0 × 10^9^/L)). Results from this meta-analysis revealed that the effect of leukocytosis was stronger in ET than in PV, and it seems exclusively related to arterial events (including recurrent events). Furthermore, the association of leukocyte count with thrombosis was confirmed in the five studies included in this meta-analysis that used time-dependent WBC measurements [12]. In a later study, by contrast, Ronner et al. found that in PV, persistent leukocytosis is associated with disease evolution but not thrombosis [28]. Altogether, to date, no definitive conclusion can be drawn [26], precluding that this variable has not been incorporated to the thrombotic risk scores. However, according to the ELN, one of the objectives of leukoreduction in these patients should be to maintain the white blood cell (WBC) count within the range of normality [29].

Finally, three meta-analyses have confirmed that patients with ET-JAK2V617F positives have up to twice more risk of thrombosis [4], possibly due to the greater degree of leukocyte and platelet activation compared to those JAK2V617F negatives [4,5]. Accordingly, in addition to cardiovascular risk factors, age and previous thrombosis, the recent IPSET-thrombosis risk assessment system for patients with ET has incorporated the JAK2V617F mutation as an independent risk variable [23] with potential clinical consequences. Based on retrospective observational studies, low-dose acetylsalicylic acid reduced incidence of venous thrombosis in JAK2V617F-mutated patients, while in CALR-mutated patients it did not affect the risk of thrombosis but was associated with a higher incidence of bleeding [30].

## 4. Role of Neutrophils in MPN Thrombosis

Classically, the pathophysiological relationship between leukocytosis and thrombosis in MPN has been explained by the increase of the cellular component and by the interaction of these cells with the endothelium and with the activated platelets [4].

**Figure 1:**
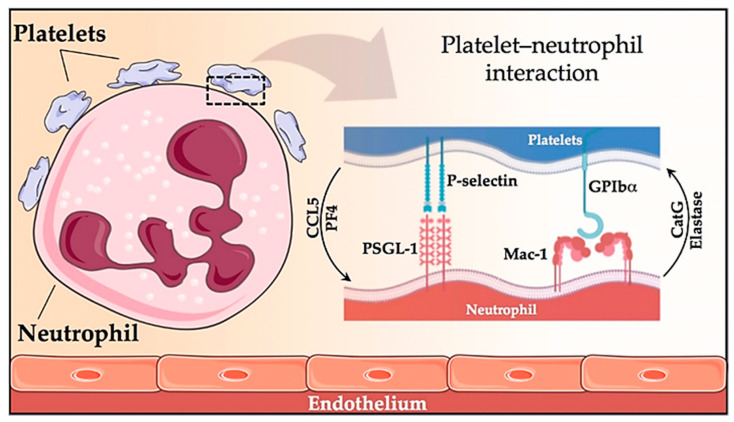
Platelet-neutrophil interaction. Representation of the two major receptor-ligand interactions in platelet-neutrophil communication, involving the P-selectin-PSGL1 and GPIbα-Mac-1 pairs; as well as the pathways by which platelets enhance leukocyte activation (by release of CCL5 and PF4) and vice versa (platelets are activated by release of elastase and cathepsin G from neutrophils).

In humans, the most abundant leukocytes in the blood are neutrophils (60–70%). Patients with MPN exhibit neutrophil activation, as evidenced by an increase in the CD11b membrane and an increase in plasma concentrations of leukocyte proteases (elastase, myeloperoxidase, cathepsin G) [4,5]. The latter causes an increase in the expression of endothelial adhesion receptors (Mac-1, PSGL-1, TREM-1L, CD14, and LAP), which favors the adhesion of these cells to the damaged vessel [4,31,32]. Neutrophils also bind to activated platelets modulating each other’s functions. Platelets enhance leukocyte activation by the release of CCL5 (RANTES) and platelet factor 4 (PF4) while conversely, neutrophils stimulate platelet activation by the release of elastase and cathepsin G (CatG) [33]. The two major receptor–ligand couples involved in the platelet–neutrophil interaction are P-selectin–PSGL1 and GPIbα–Mac-1 (Figure 1) [33]. The formation of platelet–neutrophil complexes, as well as platelet–monocyte aggregates, is well known in MPN patients [34]. JAK2V617F patients showed higher leukocyte counts and leukocyte activation (as revealed by increased membrane CD11b expression, and neutrophil-platelet aggregates) than those with JAK2 unmutated [34]. Among JAK2 unmutated patients, our group did not find differences in the parameters of platelets or leukocyte activation between mutated and unmutated patients on Calreticulin (CALR) [35].

Platelets and neutrophils interact in MPN as well as in other processes such as infection, inflammation, and thrombosis. Downstream effects of the platelet–neutrophil interaction include increased production of reactive oxygen species (ROS), increased transmigration of leukocytes over the endothelial cell lining, activation of tissue factor (TF), production of bioactive leukotrienes, and generation of neutrophil extracellular traps (NETs) [33].

## 5. Neutrophil Extracellular Traps (NETs) Formation

Neutrophils, the major innate immune cells, eliminate pathogens by phagocytosis or by releasing antimicrobial proteolytic enzymes present in their granules. In recent years, another strategy by which neutrophils kill pathogens has been identified and named NETosis [36]. NETs are extracellular structures composed by DNA and histones (nucleosomes) associated with antibacterial proteins (including myeloperoxidase, elastase, pentraxin, matrix metalloproteinase 9 (MMP9)) that entrap, immobilize and kill pathogens aiding against infections [36,37].

**Figure 2:**
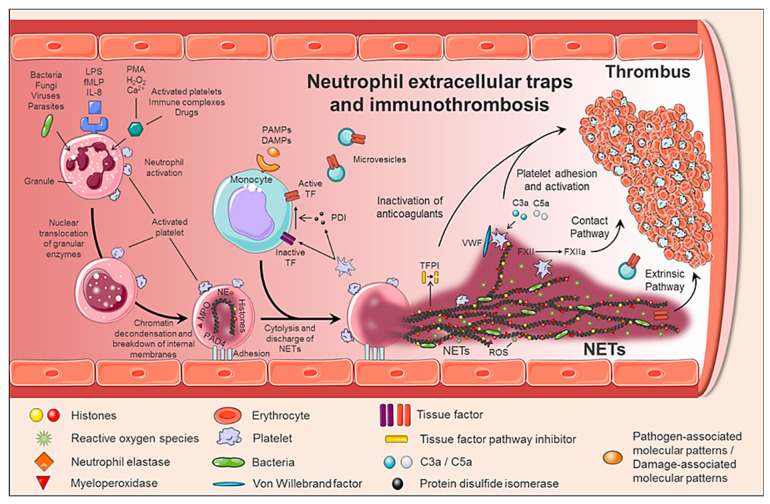
Generation of neutrophil extracellular traps (NETs) and immunothrombosisconcept. The formation of NETs is a dynamic and complex process, in which not only neutrophils are involved, but also other circulating cells such as monocytes and platelets. When activated, neutrophils adhere to the endothelium and granular enzymes (myeloperoxidase and elastase) are translocated to the nucleus, which together with the activation of PAD4 promotes the decongestion of chromatin, the loss of the lobular form of the neutrophil and the rupture of its nuclear membrane. Finally, granular proteins bound to chromatin are expelled into the extracellular space (NETs), providing a perfect structure not only to immobilize and to kill pathogens, but also to induce a pro-coagulant response.

NETs formation is a dynamic process (Figure 2). Upon activation, neutrophils adhere to the endothelium and granular enzymes (myeloperoxidase and elastase) are translocated into the nucleus. The latter, together with the activation of the enzyme peptidyl arginine deaminase 4 (PAD4) promote the de-condensation of chromatin, the loss of the lobular form of the neutrophil [38,39], and the rupture of its nuclear membrane. Granular proteins bound to chromatin are expelled into the extracellular space with or without rupture of the plasma membrane -processes called suicidal or vital NETosis, respectively. In the vital NETosis, neutrophils survive NET release and can continue to phagocytize pathogens [40,41]. The suicidal NETosis, by contrast, is considered a specific form of cellular death, dependent on the activation of nicotinamide adenine dinucleotide phosphate (NADPH) oxidase and the generation of ROS [42]. Whether one mechanism or the other is induced depends on the stimulus that triggers the process [40,41,43]: pathogens (bacteria, fungi, viruses, and protozoa) in the vital NETosis or inflammatory stimuli (LPS, IL-8, TNFα), activated platelets, auto-antibodies, or cholesterol crystals in the suicidal (or sterile) NETosis. In both vital and suicidal NETosis, PAD4-mediated histone citrullination is thought to promote NETs formation by inducing chromatin decondensation, facilitating the expulsion of chromosomal DNA [38,39]. Thus, PAD4 is essential in the NETs formation, and PAD4-deficient mice are unable to generate NETs [38,39].

Although NETs play a critical role in immune defense, excessive formation, or ineffective elimination can result in unwanted adverse effects. Therefore, its degradation is an important physiological process carried out by DNase I. Although the mechanisms involved in the clearance of NETs are not yet fully understood, macrophages also participate in the clearance of NETs by endocytic processes [37,44].

## 6. Role of NETs in Thrombotic Pathogenesis

NETs therefore not only serve as mediators of neutrophil antibacterial functions but also provide a scaffold for inducing a strong procoagulant response. Engelmann and Massberg introduce the term “immunothrombosis” to describe the link between innate immunity and thrombosis (Figure 2). It is based on the capacity of the NET to induce a procoagulant response that leads to the formation of a thrombus as a physiological defense mechanism against pathogens [45]. “Immunothrombosis is supported by immune cells and by thrombosis-related molecules” [45,46]:

- Any cell death is a potential source of free DNA in plasma, so this is a necessary but not specific finding of NETosis. Although not specific to NETosis, the presence of negative charges (DNA) causes an activation of FXII, a plasma serine protease, initiating the intrinsic pathway of coagulation. This promotes the chain activation of a series of coagulation proteins which in turn results in the formation of fibrin and ultimately the thrombus [47];
- Histones are the most abundant proteins in NETs. They are positively charged and are responsible for packaging the genetic material. It has been shown that histones 3 and 4 (H3 and H4, respectively) are able to activate platelets, favoring their aggregation and contributing to the generation of thrombin [48]. This ability of H3 and H4 to activate platelets seems to be, at least partially, dependent on the signaling pathway of TLR2 and TLR4 receptors, through the transcription factor NF-κB [48]. Alternatively, histones also contribute to thrombin activation by reducing thrombomodulin-dependent protein C activation [49];
- The granule proteases (elastase and Cathepsin G) are enzymes derived from neutrophils and the most abundant proteins in NET after histones. Elastase is located in the acidophilic granules and its function is to eliminate tissue degradation products of pathogens. In the context of thrombogenesis, it causes the degradation and inactivation of two important natural anticoagulants: tissue factor pathway inhibitor (TFPI) and antithrombin (AT) [50,51]. TFPI is the main inhibitor of the TF pathway or extrinsic pathway of coagulation whereas AT blocks thrombin formation, a key step for thrombus formation. In addition, elastase promotes platelet adhesion by facilitating exposure to von Willebrand factor (vWF). Cathepsin G hydrolyzes proteins and also helps block the activity of TFPI and enhance thrombosis by activating protease receptor 4 (PAR4) signaling pathway on platelets. Thus, it was observed that mice deficient in elastase and cathepsin G have defects in TF activation, in fibrin formation, and in thrombus stabilization [52];
- TF, through activation of the extrinsic pathway of coagulation and platelets, promotes thrombus formation. TF has been identified in NETs and it has been documented that this factor comes not only from monocytes that migrate to the inflamed area, but also from neutrophils. This finding was observed in neutrophils isolated from patients with sepsis. The autophagy has been pointed out as the mechanism by which the neutrophil captures the TF that is released during NETosis. In this sense, the TF carried by NETs is capable of stimulating thrombin generation and platelet activation in ex vivo experiments [53,54].

Altogether, NETs generate an intravascular scaffold, according to which the fibrin network facilitates the recognition, containment and destruction of pathogens [45]; the microthrombus prevents the invasion of pathogens through the circulation and generates a compartment where antimicrobial substances are concentrated for greater effectiveness; finally, the accumulation of fibrinogen or fibrin deposits promotes the recruitment of other immune cells by coordinating the immune response [45,46].

## 7. NETs in Vascular Pathology

Recently, the number of pathologies in which NETs can play a relevant role is increasing. There is an increasing amount of clinical and experimental data supporting the role of NETs in a wide variety of pathological conditions, both infectious and non-infectious [40]. Thus, the presence of NETs in autoimmune diseases, diabetes, atherosclerosis, vasculitis, and cancer has been pointed out. Furthermore, uncontrolled production of NET in blood vessels may constitute a decisive biological basis for the development of thrombotic events, including venous thrombosis, arterial thrombosis, and microvascular thrombosis [46,52,55].

Different physiopathological processes normally trigger thrombotic events, both venous and arterial, although they share common risk factors. Thus, in arterial thrombosis, the activation, aggregation, and adhesion of platelets to the endothelial wall play a very important role, ultimately leading to the formation of so-called “white” platelet-rich thrombi. In contrast, a key factor for venous thrombosis is a reduction in blood flow and activation of circulating coagulation factors, which results in ”red” thrombi due to local accumulation of large numbers of red blood cells.

The participation of NETs in arterial, venous and microvascular thrombosis has been validated both in animal models and in clinical studies [56,57,58]. Inferior vena cava and iliac vein stenosis in mice and baboons, respectively, demonstrated the presence of NETs associated with vWF within the venous thrombus and an increase of NETs markers in plasma [57,59]. In addition, the injection of extracellular histones promotes the development of DVT, while the administration of DNase I attenuates it [59]. NETs have also been identified in human venous thrombi and in plasma of patients with DVT and venous thromboembolism, being associated with increased thrombotic risk [60]. Indeed, sera and plasma from patients with primary antiphospholipid syndrome (PAPS), who carry a markedly increased risk of thrombotic events and pregnancy loss, showed elevated levels of NETs, as compared to healthy volunteers [61]. Specifically, administration of IgG from these patients accelerates venous thrombosis in a flow restriction murine model, a phenotype that associates with human IgG binding to the neutrophil surface and with a expanded infiltration of NETs into the thrombi themselves [62].

Moreover, recent studies have shown how NETs contribute to the initiation and progression of atherosclerotic lesions and arterial thrombus growth [63]. Thus, in a murine model of atherosclerosis, PAD4 inhibition was able to prevent the formation of NETs, decrease the size of the atherosclerotic lesion, and delay carotid artery thrombosis [64]. Another work performed on ApoE^−/−^ mice showed that cholesterol crystals (sterile stimulus) have the capacity to generate NETs that activated macrophages, amplifying cell recruitment in the lesion area [65]. In humans, the presence of NETs has been associated with coronary atherosclerosis and myocardial infarction [66]. 

Finally, patients with sepsis and/or disseminated intravascular coagulation have elevated TF levels in monocytes, leukocyte-platelet aggregates, and increased levels of NETs markers [67,68].

## 8. Role of NETs in Myeloproliferative Neoplasms

As previously mentioned, NETs appear on both infectious and non-infectious diseases, e.g., autoimmune disease or cancer, under the stimulus of cytokines (TNFα and IL-8) secreted by the neoplasm clone itself [69] or by activated platelets [70,71,72]. When platelets are activated, P-selectin is translocated to the membrane from α-granules. P-selectin, both cellular and soluble, promotes NETosis through binding to PSGL-1. The process can be inhibited by blocking either P-selectin or PSGL-1. Indeed, activated platelets from P-selectin null mice were unable to trigger NETs, whereas neutrophils from mice engineered to overproduce soluble P-selectin had excessive agonist-induced NETs formation, suggesting that the P-selectin/PSGL-1 axis is a potential therapeutic target [73].

Although it is a physiological process, uncontrolled production of NETs may constitute the basis for the development of thrombotic disorders [46,52]. In a recent prospective observational cohort study with nearly 1000 cancer patients and two years of follow-up, citrullinated H3 (citH3), a biomarker of NET formation, predicted the risk of venous thromboembolism. Thus, citH3 levels had a magnitude of association with venous thromboembolism risk comparable to D-Dimer or soluble P-selectin [72].

Specifically, three studies have evaluated whether NETs contribute to the procoagulant state in MPN patients [74,75,76]. Although it seems obvious that the percentage of neutrophils with increased levels of ROS is higher in patients with MPN than controls [75,76], it is not clear if under baseline conditions, i.e., without stimulation, they produce more NETs. Whereas Guy et al. showed that unstimulated neutrophils from patients with MPN, ex vivo, produced more NETs than control subjects [76], Oyarzún et al. and Wolach et al., in two independent studies did not find enhanced NETosis by unstimulated JAK2V617F neutrophils [74,75]. These contradictory results have been attributed to the fact that in the last two cohorts of patients [74,75], most of the patients were receiving JAK inhibitors or cytoreductive treatment at the time of inclusion in the study. Other potential biases that may explain these contradictory results could be derived from the small number of patients included for this purpose in 2 out of the 3 studies (*n* = 19 and 32 patients in Oyarzún et al. and Wolach et al. studies, respectively), and the enrichment of patients with previous thrombosis in the third of these studies (26 out of 52, 50% of patients in Guy’s cohort) [76]. One additional explanation is the non-standardized assays used to assess NETosis [74,75,76]. In fact, there are still no methods to assess and determine NETosis in a reproducible and objective manner [77].

Regarding the formation of NETs ex vivo, under stimulation, the results are also contradictory. Ex vivo stimulation of neutrophils with ionomycin, caused an increase in NET formation (citH3 expression) in both JAK2V617F human and mouse neutrophils [74]. By contrast, another study did not find NETs production after stimulation with IL-8, or TNFα; and with a stronger NETs inducer, such as PMA, MPN cells showed defective NETosis [75].

Moreover, in two independent cohorts of patients with MPN, the evaluation of plasmatic biomarkers of NETosis has shown an increase in the concentration of free plasma DNA [76] and elevated levels of circulating nucleosomes [75], another DNA marker. However, free plasma DNA or nucleosomes are not specific markers of NETs, they can be originated also from other forms of cell death, such as apoptosis or necrosis. More specific markers of NETs combine measurement of DNA (nucleosomes, histones, or free DNA) with a specific enzyme (myeloperoxidase o elastase) from neutrophils. While Oyarzún et al. did not find higher levels of histone-MPO in MPN patients as compared to healthy donors [75], Guy et al. showed a significant increase in MPO-DNA concentration in patients with MPN at the time of presentation compared to controls [76]. Importantly, MPO-DNA levels were higher in MPN patients with previous thrombosis, especially with splenic thrombosis, positioning itself as a biomarker of thrombosis in patients with MPN [76].

In regards to the effect of cytoreductors on NET markers, ruxolitinib abrogates NETs formation ex vivo (in neutrophils from patients receiving the JAK1/2 inhibitor) and, in vivo, decreasing thrombosis in JAK2V617F mice [74]. Oyarzún et al. showed that both hydroxyurea and ruxolitinib decrease the concentration of nucleosomes [75]. By contrast, Guy et al. (in 10 patients) did not find that treatments were associated with a decrease of free DNA or MPO-DNA complexes, despite the normalization of neutrophils counts [76].

In JAK2V617F/WT; Vav-Cre mice, with heterozygous expression of the JAK2V617F allele in hematopoietic cells (JAK2V617F), Wolach et al. demonstrated an increased lung thrombi formation. To further explore the role of NETosis in MPN thrombosis, these authors investigated the development of thrombosis in an experimental model of NET-dependent thrombosis in JAK2V617F mice [74]. Two hours after partial ligation of the inferior vena cava, 45% of the JAK2V617F mice developed thrombosis while none of the JAK2 wild type (JAK2WT) mice. The treatment during 72 h with ruxolitinib reduced the range of thrombosis to levels comparable to JAK2WT mice and decreased the content of neutrophils and citH3 within the thrombi [74]. The same group demonstrated that both JAK2V617F-driven NET formation and thrombosis are dependent on PAD4; which was found overexpressed in neutrophils from patients with PV harboring JAK2V617F [74].

Finally, elegant murine studies in two different mouse models, one of them with the expression of JAK2V67F in all hematopoietic cells, and, the other one with the expression of JAK2V67F only in neutrophils, demonstrated that JAK2V617F neutrophils alone are not enough to promote NETosis and thrombosis, and that they need to cooperate with platelets to induce NETs formation [78].

## 9. New Therapeutic Opportunities to Prevent Thrombosis in MPN

Thrombogenesis in MPN involves multiple cellular mechanisms, including platelet and leukocyte activation and neutrophil extracellular trap formation. In this framework, there is increasing interest in exploring antithrombotic therapies that target these processes. Thus, decondensed chromatin of NETs is sensitive to DNase I, the predominant nuclease in plasma. In patients with acute coronary syndrome, DNase I accelerated, ex vivo, tPA-mediated thrombolysis on coronary thrombi. Moreover, DNase I breaks down the extracellular DNA present in the sputum of patients with cystic fibrosis and it is a safe drug. In fact, FDA/EMA-approved its use for this disease [79]. Prevention of NETs formation could be another potential therapeutic approach. In this regard, PAD4 inhibitors have been developed and are currently being validated [80]. In fact, a novel PAD4-specific inhibitor, BMS-P5, developed by Bristol-Myers Squibb, blocks, in vitro, multiple myeloma cells-induced NETs formation, and in vivo, in a syngeneic mouse model of multiple myeloma, BMS-P5 delays disease progression [81]. Furthermore, since NETs have been linked to the pathogenesis of COVID-19 associated respiratory distress syndrome, BMS-P5 has been recently proposed as a candidate drug target for SARS-CoV-2-induced acute lung injury [82].

N-acetylcysteine (NAC) is an agent with an antioxidant and mucolytic effect. It works by increasing the level of glutathione, free radical scavenging, and reducing disulfide bonds. It is currently used for acetaminophen overdose, contrast nephropathy prophylaxis, and as a mucolytic agent in cystic fibrosis. Acetylcysteine is also indicated as an adjuvant treatment in respiratory processes with excessive or thick mucous secretion such as bronchitis, chronic obstructive pulmonary disease, emphysema, and atelectasis due to mucous obstruction. Moreover, in a mouse model of venous thrombosis in JAK2V617F mice, N-acetylcysteine reduced thrombus formation and the thrombin-induced platelet-leukocyte aggregate formation. Ex vivo, NAC reduced NETs formation in stimulated neutrophils from patients with MPN [83], postulating it as a potential agent to reduce thrombosis in these patients.

Finally, Edelmann et al. recently demonstrated that neutrophils expressing JAK2-V617F have increased activation of β1and β2-integrin, resulting in an increased adhesion to VCAM and ICAM1 on the vascular endothelium and enhanced thrombus formation. Importantly, antibodies targeting β1and β2-integrin reduce neutrophil adhesion, resulting in decreased thrombus formation [84].

## 10. Conclusions

In summary, recent studies demonstrated the participation of the neutrophil extracellular trap in thrombotic pathology in diseases with both infectious and non-infectious components. In this line, the presence of neutrophil extracellular tramp has been documented in autoimmune diseases, diabetes, atherosclerosis, vasculitis, and cancer. In addition, as mentioned above, uncontrolled production of NETs in blood vessels may constitute a decisive biological basis for the development of thrombotic disorders, including venous thrombosis, arterial thrombosis, and microvascular thrombosis. If we focus on myeloproliferative neoplasms, thrombotic complications represent an important clinical problem due to their high impact in morbidity, the complexity of their management and their associated mortality. Although very few, there are very recent works suggesting that increased formation of NETs promotes thrombosis in the setting of MPN patients. Thus, an association between NETosis markers and the occurrence of thrombosis in MPN patients has been recently suggested. Future studies are needed to support this association, and to demonstrate whether they cannot only be used as pathogenic markers but also as candidate drug target for thrombotic disease in MPN. 

## Glossary

MPN	Myeloproliferative neoplasm
PV	Polycythemia vera
ET	Essential thrombocythemia
MF	Myelofibrosis
HSC	Hematopoietic stem cell
BM	Bone marrow
JAK	Janus kinase
STAT	Signal transducer and activator of transcription
ECLAP	European collaboration on low-dose aspirin in polycythemia vera
IWG-MRT	International Working Group for MPN Research and Treatment
CYTO-PV	Cytoreductive therapy in PV
AVWS	Acquired von Willebrand syndrome
ELN	European Leukemia Net
WBC	White blood cell
WHO	World Health Organization
IPSET	International prognostic score for thrombosis in ET
Mac-1	Macrophage-1 antigen
PSGL-1	P-selectin glycoprotein ligand 1
TREM1	Triggering receptor expressed on myeloid cells 1
LAP	Latency-associated peptide
CCL5	C-C motif chemokine 5
PF4	Platelet factor 4
GPIbα	PlateletglycoproteinIb alpha chain
CALR	Calreticulin
ROS	Reactive oxygen species
TF	Tissue factor
NETs	Neutrophil extracellular traps
DNA	Deoxyribonucleic acid
MMP9	Matrix metalloproteinase 9
PAD4	Peptidyl arginine deaminase 4
LPS	Lipopolysaccharide
NADPH	nicotinamide adenine dinucleotide phosphate
IL-8	Interleukin 8
TNFα	Tumor necrosis factor alpha
FXII	Factor XII
TLR2	Toll-like receptor 2
TLR4	Toll-like receptor 4
NF-κB	Nuclear factor kappa-light-chain-enhancer of activated B cells
TFPI	Tissue factor pathway inhibitor
vWF	von Willebrand factor
AT	Antithrombin
PAR4	Protease activated receptor 4
DVT	Deep vein thrombosis
ApoE	Apolipoprotein E
citH3	Citrullinated histone H3
PMA	Phorbol 12-myristate 13-acetate
MPO	Myeloperoxidase
WT	Wild type
tPA	Tissue plasminogen activator
FDA	Food and Drug Administration
EMA	European Medicines Agency
NAC	N-acetylcysteine
VCAM1	Vascular cell adhesión molecule 1
ICAM1	Intercellular adhesion molecule 1
fMLP	N-Formylmethionyl-leucyl-phenylalanine
H_2_O_2_	Hydrogen peroxide
Ca^2+^	Calcium ion
PAMPs	Pathogen-associated molecular patterns
DAMPs	Damage-associated molecular patterns
CatG	Cathepsin G
PDI	Protein disulfide isomerase
